# Erratum

**DOI:** 10.1590/2177-6709.24.3.113.err

**Published:** 2019

ERRATUM: AN INTERVIEW WITH KEVIN O’BRIEN

In the INTERVIEW “An interview with Kevin O’Brien”, with DOI 10.1590/2177-6709.22.5.018-021.int, published in Dental Press J Orthod. 2017 Sept-Oct;22(5):18-21, had in authorship only “Kevin O’Brien” as author. 

Now it should have “**Kevin O’Brien, Klaus Barretto Lopes, José Augusto Mendes Miguel, Jonathan Sandler, Ki Beom Kim**” as authors of the interview.

ERRATUM: AN INTERVIEW WITH MARIO POLO

In the INTERVIEW “An interview with Mario Polo”, with DOI 10.1590/2177-6709.22.6.014-024.int, published in Dental Press J Orthod. 2017 Nov-Dec;22(6):14-24, had in authorship only “Mario Polo” as author. 

Now it should have “**Mario Polo, Flavia Artese, Mayra Seixas, Víctor L. Acevedo Rivera, Nelson Mucha**” as authors of the interview.

ERRATUM: AN INTERVIEW WITH CHRIS CHANG

In the INTERVIEW “An interview with Chris Chang”, with DOI 10.1590/2177-6709.23.1.018-021.int, published in Dental Press J Orthod. 2018 Jan-Feb;23(1):18-21, had in authorship only “Chris Chang” as author. 

Now it should have “**Chris Chang, Marcio Rodrigues de Almeida, Matheus Pithon, Weber Ursi**” as authors of the interview.

ERRATUM: AN INTERVIEW WITH AMBROSINA MICHELOTTI

In the INTERVIEW “An interview with Ambrosina Michelotti”, with DOI 10.1590/2177-6709.23.2.022-029.int, published in Dental Press J Orthod. 2018 Mar-Apr;23(2):22-9, had in authorship only “Ambrosina Michelotti” as author.

Now it should have “**Ambrosina Michelotti, Ana Cláudia de Castro Ferreira Conti, Ricardo de Souza Tesch, Guilherme de Araújo Almeida, Bruno D’Aurea Furquim, Daniela Aparecida de Godoi Gonçalves**” as authors of the interview.

ERRATUM: AN INTERVIEW WITH EWA CZOCHROWSKA

In the INTERVIEW “An interview with Ewa Czochrowska”, with DOI 10.1590/2177-6709.23.3.014-023.int, published in Dental Press J Orthod. 2018 May-June;23(3):14-23, had in authorship only “Ewa Czochrowska” as author.

Now it should have “**Ewa Czochrowska, Guilherme Thiesen, Perpétua Freitas, Eduardo Franzotti Sant’Anna, Ildeu Andrade Jr.**” as authors of the interview.

ERRATUM: GLOBAL DISTRIBUTION OF MALOCCLUSION TRAITS: A SYSTEMATIC REVIEW

In the article “Global distribution of malocclusion traits: A systematic review”, with DOI 10.1590/2177-6709.23.6.40.e1-10.onl, published at Dental Press J Orthod. 2018 Nov-Dec;23(6):40.e1-10, in “Figure 1: Flowchart of the literature selection process.”, where it is written:

“Records identified through database searching (n=1969)” 

Should be written:

“Records identified through database searching (n=2969)

